# Effect of Phorate on the Development of Hyperglycaemia in Mouse and Resistance Genes in Intestinal Microbiota

**DOI:** 10.3390/antibiotics11111584

**Published:** 2022-11-09

**Authors:** Tingting Cao, Yajie Guo, Dan Wang, Zhiyang Liu, Suli Huang, Changfeng Peng, Shaolin Wang, Yang Wang, Qi Lu, Fan Xiao, Zhaoyi Liang, Sijia Zheng, Jianzhong Shen, Yongning Wu, Ziquan Lv, Yuebin Ke

**Affiliations:** 1 Shenzhen Center for Disease Control and Prevention, Shenzhen 518055, China; 2 The Eighth Affiliated Hospital, Sun Yat-Sen University, Shenzhen 518033, China; 3 School of Public Health, Southern Medical University, Guangzhou 510515, China; 4 College of Veterinary Medicine, China Agricultural University, Beijing 100091, China; 5 Food Safety Research Unit (2019RU014), Chinese Academy of Medical Science, NHC Key Laboratory of Food Safety Risk Assessment, China National Center for Food Safety Risk Assessment, Beijing 100021, China

**Keywords:** phorate, hyperglycaemia, intestinal microbiota, resistance genes

## Abstract

Phorate is a systemic, broad-spectrum organophosphorus insecticide. Although it is commonly used worldwide, phorate, like other pesticides, not only causes environmental pollution but also poses serious threats to human and animal health. Herein, we measured the blood glucose concentrations of high-fat-diet-fed mice exposed to various concentrations of phorate (0, 0.005, 0.05, or 0.5 mg/kg); we also assessed the blood glucose concentrations of high-fat-diet-fed mice exposed to phorate; we also assessed the distribution characteristics of the resistance genes in the intestinal microbiota of these mice. We found that 0.005 and 0.5 mg/kg of phorate induced obvious hyperglycaemia in the high-fat-diet-fed mice. Exposure to phorate markedly reduced the abundance of *Akkermansia muciniphila* in the mouse intestine. The resistance genes *vanRG, tetW/N/W, acrD*, and *evgS* were significantly upregulated in the test group compared with the control group. Efflux pumping was the primary mechanism of drug resistance in the *Firmicutes, Proteobacteria, Bacteroidetes, Verrucomicrobia, Synergistetes, Spirochaetes*, and *Actinobacteria* found in the mouse intestine. Our findings indicate that changes in the abundance of the intestinal microbiota are closely related to the presence of antibiotic-resistant bacteria in the intestinal tract and the metabolic health of the host.

## 1. Introduction

Organophosphorus (OP) compounds are widely used in China and abroad to protect crops from insects [1]. OP pesticides generally work by irreversibly inhibiting the enzyme acetylcholinesterase [2]. Phorate is a systemic, broad-spectrum OP insecticide and is commonly used in the agricultural sector to control sucking and chewing pests, leaf hoppers, and mites. Phorate is primarily available as a granular formulation that can be applied by banding when planting a crop or by directly placing it in-furrow with a seed [3]. However, the use of OP pesticides leads to varying degrees of environmental pollution, which can originate from various sources. Studies have reported that phorate is found in vegetables (tomatoes, 2.0–98.5 µg/kg; aubergines, 1.0–20.7 µg/kg) and fruits (apples, 1.5–45.1 µg/kg; grapes, 3.4 µg/kg; pears, 1.4–26.4 µg/kg) [4]. The estimated daily intake of phorate ranges from 0.1 to 0.47 µg/kg bw/day in the general population and from 0.18 to 43.73 µg/kg bw/day in farmers [5]. These numbers have raised concerns regarding human exposure to phorate.

Phorate undergoes a P450-mediated desulphurisation reaction to produce oxon metabolites; its primary mechanism of acute toxicity is acetylcholinesterase inhibition mediated mainly by the oxon metabolite phorate–oxon [6]. It remains unknown whether animals can develop resistance after exposure to phorate. Nevertheless, the emergence of resistance is thought to be linked to the use of antibiotics, which pose a threat to human and animal health [7]. In humans and animals, the gut is the key reservoir of microbial communities, comprising both commensal and pathogenic bacteria [8]. A growing number of studies have found that because the gut microbiota is frequently exposed to exogenous antibiotics from drugs or from the food chain, it possesses multiple drug-resistance genes [9]. Moreover, long-term exposure of humans and animals to antibiotics leads to the enrichment of intestinal drug-resistance genes.

A recent review of comprehensive studies on intestinal microbiota and antibiotic resistance conducted in a large human cohort in China found that the antibiotic resistance of the intestinal microbiota is closely associated with faecal metabolites and the host’s metabolic health [10]. Antibiotic-resistance gene diversity is associated with a higher risk of type 2 diabetes (T2D). Moreover, a study reported that the pesticide chlorpyrifos impaired mitochondrial function and diet-induced thermogenesis in brown adipose tissue (BAT) and promoted increased obesity, non-alcoholic fatty liver disease (NAFLD), and insulin resistance in high-fat-diet-fed mice [11]. However, to date, no studies have assessed the effects of phorate on glucose metabolism.

To explore the hazards of pesticide residues with respect to host metabolic health and the correlation between the production of resistance genes and glucose metabolism, in this study, we measured the blood glucose concentrations and assessed the distribution characteristics of antibiotic-resistance genes in the intestinal microbiota of HFD-fed mice exposed to phorate.

## 2. Results

### 2.1. Phorate Exposure Elevated the Blood Glucose Concentrations in HFD-Fed Mice

**Figure 1:**
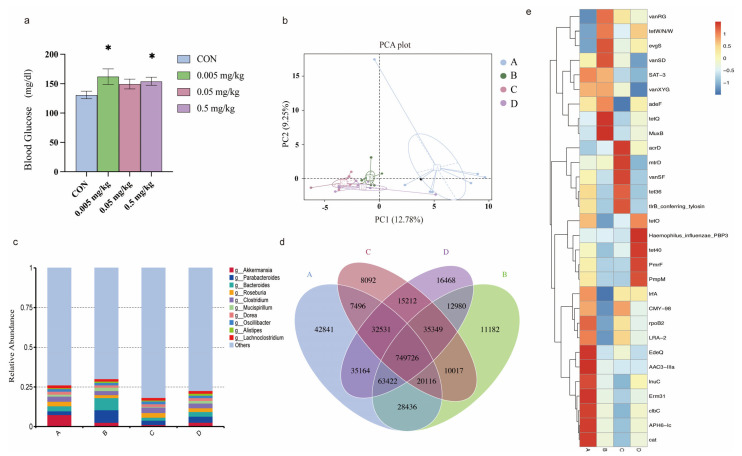
(**a**) Effect of phorate on the blood glucose concentrations of mice after 35 days of exposure. (**b**) PCA plots of the different genera. The abscissa is the first principal component, whereas the ordinate is the second principal component. Both percentages represent the contribution of the second principal component to the sample differences. Each point in the figure represents a sample, and samples of the same colour belong to the same group. (**c**) Relative-abundance bar chart of the genus-annotation results of each sample at different classification levels; the top 10 genera with the largest relative abundances in each group are indicated, whereas the remaining genera are grouped as ‘others’. (**d**) Venn diagrams showing the numbers of unique and common genes in the mouse intestinal tract. (**e**) Distribution of resistance genes in the phorate-exposed groups. *: *p* < 0.05, as assayed by two-tailed Student’s t-test or one-way ANOVA followed by Student-Newman-Keuls test.

To determine the effects of phorate on glucose metabolism, 7–8-week-old male HFD-fed C57Bl/6j mice were exposed to 0, 0.005, 0.05, and 0.5 mg/kg bw/day of phorate by oral gavage each day for 5 weeks. We then measured the blood glucose concentrations of the mice in the natural state (Figure 1a). The blood glucose concentrations of the mice exposed to phorate, especially the 0.005 and 0.5 mg/kg bw/day doses, were significantly higher than those of the control group (*p* < 0.05).

### 2.2. Effects of Phorate on the Beta Diversity of the Gut Microbiota in HFD-Fed Mice

Principal component analysis was used to study the extent of intergroup similarity or heterogeneity in terms of community structure. A shorter distance between samples indicates a greater similarity in community structure and vice versa. The community similarity was measured based on phylogenetic relatedness using unweighted UniFrac in a principal component analysis plot (Figure 1b). PC1 and PC2, the first two principal components, explain 12.78% and 9.25% of the data variation, respectively, clearly separating each community. Samples of the same group are represented by the same colour. The A group (control group) and the B, C, and D groups (0.005, 0.05, and 0.5 mg/kg group) are clearly separated, indicating that the gut microbiota structure had discernibly changed after exposure to phorate.

### 2.3. Relative Abundance of the Intestinal Microbiota in Mice Exposed to Phorate

The intergroup analysis identified the enrichment of specific genera, indicated by significant intergroup differences in abundance. Gut microbiota genera having a relative abundance of 0.01% in at least one group were scrutinized. Genera with a relative abundance of >0.01% of the intestinal microbiota genera in each group were selected. Figure 1C shows the abundances of the top 10 bacterial genera. *Akkermansia* was the dominant genus in the control group (7.43%), with the other classifications ignored. However, the abundance of *Akkermansia* was decreased in the phorate-exposed groups (B: 2.54%, C: 2.76%, D: 3.82%). The abundance of Parabacteroides (B: 7.99%, C: 2.76%, D: 2.56%) and that of *Alistipes* (B: 0.87%, C: 0.55%, D: 1.24%) were increased in the phorate-exposed groups compared with the control group (*Parabacteroides*: 2.38%, *Alistipes*: 0.53%).

### 2.4. Effects of Phorate on Intestinal Microbiota Gene Numbers

A Venn diagram was drawn to investigate the distribution of gene numbers among the designated groups and to analyse the common and unique information of the genes in the different groups. Overall, 749,726 genes were shared among the four groups (Figure 1D). In total, 42,841 genes were unique to the control group. In comparison, 11,182 unique genes were found in the 0.005 mg/kg group, 8092 in the 0.05 mg/kg group, and 16,468 in the 0.5 mg/kg group. Thus, significant differences were found in the microbial compositions of the four groups. Moreover, exposure to phorate reduced the number of intestinal microbiota genes.

### 2.5. Composition of Intestinal Antibiotic-Resistance Genes after Phorate Exposure

After analysing the drug-resistance genes in the intestinal microbiota in the four groups of mice, the 30 genes with the most obvious expression differences were subjected to further analysis (Figure 1E). The resistance genes *vanRG*, *tetW/N/W*, *acrD*, and *evgS* were significantly upregulated in the phorate-exposed groups compared with the control group. However, the resistance genes *IrfA*, *CMY-98*, *rpoB2*, *LRA-2*, *EdeQ*, *AAC3-IIa*, *InuC*, *Erm31*, *clbC*, *APH6-Ic*, and *cat*-resistance genes were significantly downregulated regulated in the phorate-exposed groups.

### 2.6. Relationship between the Resistance Gene Mechanisms and the Intestinal Microbiota Composition

**Figure 2:**
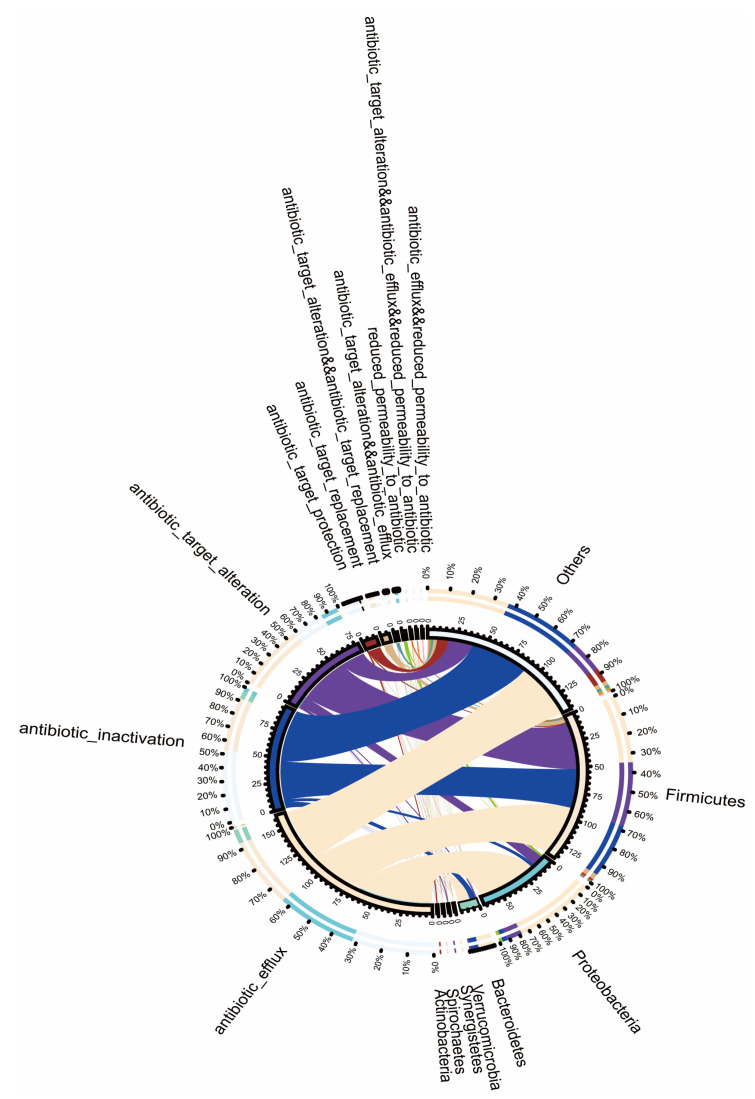
Circular diagram outlining the resistance mechanisms and genera. The circle is divided into two parts, with the genus information at the gate level on the right and the resistance mechanism-related information on the left. The different colours in the inner circle indicate the resistance mechanisms for different genera and resistances, and the scale denotes the number of genes. The left side indicates the sum of the number of resistance genes in the genera that contain the corresponding resistance mechanism, and the right side denotes the sum of the number of resistance genes contained in the genera with different resistance mechanisms. The left side of the outer circle denotes the relative proportion of the resistance genes in each genus to the resistance genes of its resistance mechanism, whereas the right side of the outer circle denotes the relative proportion of the resistance genes in each resistance mechanism to the resistance genes of its genus.

Figure 2 shows that the most abundant phyla in the mouse intestinal microbiota were *Firmicutes* and *Proteobacteria*. The abundance of *Firmicutes* is mainly attributable to the following three mechanisms of drug resistance: efflux pumping, inactivation, and target alternation. Meanwhile, efflux pumping is the main mechanism of drug resistance in *Proteobacteria*. *Bacteroidetes*, *Verrucomicrobia*, *Synergistetes*, *Spirochaetes*, and *Actinobacteria* were also widely distributed in the intestinal microbiota of the mice. Moreover, target protection, replacement, and other mechanisms exhibited by the above-mentioned strains affect the development of drug resistance by the intestinal microbiota.

## 3. Discussion

Phorate is a systemic, broad-spectrum OP insecticide. In this study, for the first time, we found that phorate has a hyperglycaemic effect on HFD-fed mice. Diabetes is reportedly induced by environmental and genetic causes, and increasing evidence has shown that the use of global pesticides has increased the risk of obesity and T2D [12]. A previous study reported that the increased risk of diabetes in humans may be related to the use of phorate [13]. The body weight changes of the four groups of mice were recorded during the whole experiment. However, we did not find a significant difference in body weight between the phorate groups and the control group (data not shown). We felt that there was probably no relationship between weight and blood glucose levels in this study. Among 13,637 farmers’ wives who were exposed to phorate, 688 (5%) were found to have diabetes over a 10-year follow-up period. This finding is consistent with our hyperglycaemia results in mice. However, our mouse experiments clearly demonstrated a dose-dependent relationship between phorate and hyperglycaemia. Mice exposed to 0.005 and 0.5 mg/kg phorate had obvious hyperglycaemia. Phorate is a potential risk factor for human health, and the acceptable daily intake recommend by the WHO is 0.0005 mg/kg bw/day [14]. Occupational exposure of farmers to phorate increases their daily intake of pesticides, with a median exposure of 0.69 μg/kg bw/day, the equivalent of which is 0.006279 mg/kg bw/day in mice [5]. Phorate at concentrations of 0.25–2 μg/mL can lead to the splitting and mutation of DNA as well as DNA loss in human lymphocytes [15]. However, the regulatory roles of phorate in glucose metabolism remain unclear.

Upon entry through oral gavage, phorate inevitably affects the intestinal microbiota of mice. The gut microbiome is closely associated with the occurrence and development of chronic diseases [16]. The gut microbiota engages in symbiotic relationships and regulates various metabolic functions, including intestinal barrier homeostasis and glucose homeostasis [17,18]. Preclinical and clinical studies have shown that the abundance of *A*. *muciniphila* is associated with the development of metabolic disorders, including obesity and T2D [19,20]. *A*. *muciniphila* secretes a glucagon-like peptide-1-inducing protein to improve glucose homeostasis and regulate metabolic diseases in mice [21]. Similarly, in the present study, we found that the abundance of *A*. *muciniphila* was significantly reduced after exposure to phorate, and this may have led to the observed increases in blood glucose concentrations. However, the abundances of *Parabacteroides* and *Alistipes* were increased in the phorate-exposed groups compared with those in the control group. *Parabacteroides* can accelerate the development of diabetes in non-obese diabetic mice as well as increase the macrophage, dendritic cell, and destructive CD8+ T cell levels and reduce the Treg cell levels [22]. Feeding on HFDs increases the abundance of *Alistipes* [23]. The enrichment of anti-inflammatory bacteria in *Alistipes* can improve its glucose tolerance and insulin sensitivity [24]. Our results were consistent with those of previous studies and suggested that the changes in the intestinal microbiota induced by phorate were closely associated with glucose metabolism.

Interestingly, we found that phorate may also contribute to the development of resistance genes in the intestinal microbiota as well as the development of hyperglycaemia in mice. According to a recent study [10] that analysed the metagenomic landscape of intestinal antibiotic-resistant microorganisms in a large multiomics human cohort (*n* = 1210) study, a significant overall change was observed in the intestinal antibiotic-resistance structure of the healthy, prediabetic, and T2D groups. The study reported that the levels of *vanRG*, *tetW/N/W*, *acrD*, and *evgS* were significantly upregulated after exposure to phorate. *vanX* present in vancomycin-resistant genes is reportedly associated with the risk of T2D [10]. *AcrD*, which is paralogous to *AcrB*—which belongs to the RND family of transporters—confers resistance to tetracycline, novobiocin, nalidixic acid, norfloxacin, sodium dodecyl sulfate, and aminoglycosides [25]. We found that hyperglycaemic mice carried the vancomycin-resistance gene *vanRG*, the tetracycline-resistance gene *tetW/N/W*, and the multidrug-resistance genes *acrD* and *evgS*; these genes have not been previously reported in mice. We believe that the increase in the abundances of these resistance genes is attributable to the use of phorate.

For the first time, we found that phorate can lead to the production of drug-resistance genes in the intestinal microbiota. Phorate can also lead to the corresponding drug resistance in bacteria. For instance, tetracycline efflux transporters are a major facilitator of the antibiotic efflux pumps of the aminoglycoside gene superfamily [26]. We found that efflux pumps were the main mechanism of drug resistance in *Firmicutes*, *Proteobacteria*, *Bacteroidetes*, *Verrucomicrobia*, *Synergistetes*, *Spirochaetes*, and *Actinobacteria*. Notably, *Firmicutes*, *Proteobacteria*, and *Bacteroidetes* have been found to be dominant in the microbiota of mice and humans [27]. Therefore, we suspect that phorate causes hyperglycaemia in mice primarily by causing the dominant bacteria in the intestine to produce efflux pumps. Some studies have reported that the gut antibiotic resistome may change earlier than the gut microbiota during T2D progression and/or that changes in the gut antibiotic resistome are more sensitive to the development of T2D [10]. Herein, we propose that phorate can cause hyperglycaemia in mice by influencing the abundance of the intestinal microbiota and by modulating or altering the expression of drug-resistance genes.

## 4. Materials and Methods

### 4.1. Chemicals

Phorate was obtained from Tianjin Alta Scientific Co., Ltd. (Tianjin, China) (purity quotient of ≥99%, product no. 298-02-2). Pure corn oil was purchased from Sigma-Aldrich (100%, product no. C116023; Sigma-Aldrich (Shanghai, China) Trading Co, Ltd, Shanghai, China). The mice were fed with a HFD (60 kcal% fat; Cat# D12492; Research Diets Inc., New Brunswick, NJ, USA).

### 4.2. Animals and Treatments

A total of 28 male C57Bl/6j mice aged 7–8 weeks were obtained from Beijing Vital River Laboratory Animal Technology Co., Ltd. (Beijing, China). The mice were randomly divided into four groups (control, 0.005 mg/kg, 0.05 mg/kg, and 0.5 mg/kg) and fed an HFD. Based on their group, the mice were treated with different doses of phorate or corn oil (control) daily for 5 weeks consecutively. Phorate dissolves well in corn oil and was administered by gavage. All the mice were housed under a 12 h light/dark cycle at 22–25 °C and were provided free access to drinking water and food, except when the food had to be withdrawn for experimental purposes. All of the animal experiments were performed according to the guidelines of Shenzhen TopBiotech Co., Ltd (Shenzhen, China). (TOP-IACUC-2021-0083).

### 4.3. Blood Glucose Concentrations

Blood glucose concentrations were determined using a FreeStyle Optium Neo meter (Abbott, Shanghai, China) at the fifth week of the treatment period. Blood samples were collected from mice through a small cut made at the tip of the tail.

### 4.4. Metagenomic Sequencing

Using the QIAamp DNA Stool Kit (Qiagen, Gaithersburg, MD, USA), genomic DNA was extracted from the caecum contents. A Thermo NanoDrop One (Thermo Fisher Scientific Co., Ltd, Waltham, USA) was used to detect the purity and concentration of the extracted DNA (*n* = 28 C57Bl/6j mice fed an HFD). We used the paired-end sequencing mode of the Illumina HiSeq sequencing platform (Novogene Company Limited Co., Ltd, Tianjin, China) for high-throughput sequencing of multiple samples. The raw data obtained using this platform were pre-processed using Readfq to acquire clean data for subsequent analyses. Bioinformatic analysis of the sequencing data was conducted using the Quantitative Insights into Microbial Ecology software. Low-quality reads, barcodes, and primers as well as chimera sequences were eliminated using the UCHIME software (version 4.2, http://drive5.com/usearch/manual/uchime_algo.html) with the relevant algorithm, and the effective tags were obtained. Clean data were obtained after pre-processing, and the MEGAHIT assembly software (version 1.0.4-beta) was used for assembly analysis. After quality control of each sample, the clean data were compared with the scaftigs after assembly of each sample using the Bowtie2 software (version 2.2.4, http://bowtie-bio.sourceforge.net/bowtie2/index.shtml). For the scaftigs generated upon single-sample assembly, fragments <500 bp were filtered out, and statistical analysis and subsequent gene prediction were conducted. Starting from the scaftigs (≥500 bp) of each sample, MetaGeneMark (version 3.05, http://topaz.gatech.edu/GeneMark/) was used for open reading frame prediction, and hits with <100 nucleotides were filtered out based on the prediction results. For the open reading frame prediction results of each sample assembly, the CD-HIT software (version 4.5.8, http://www.bioinformatics.org/cd-hit/) was used to eliminate redundancies to obtain a non-redundant initial gene catalogue. By default, 95% identity and 90% coverage were maintained for clustering, and the longest sequence was selected as the representative sequence. Based on the abundance information of each gene in each sample in the gene catalogue, basic information statistics, core pan-gene analysis, correlation analysis between samples, and gene-number Wayne diagram analysis were conducted. The 28 gut microbial metagenome sequence data that support the findings are available in the SRA under the NCBI BioProject ID PRJNA892724.

### 4.5. Annotation of Resistance Genes

Resistance gene identifier (RGI) software (version 6.0.0, https://card.mcmaster.ca/analyze/rgi) in the Comprehensive Antibiotic Resistance Database (v2.0.1) was used to compare unigenes with the CARD data (RGI built-in Blastp); bitcore value comparison was performed to score the results [28]. The relative abundance of each Antibiotic Resistance Ontology was calculated based on the comparison results.

### 4.6. Statistical Analysis

Based on the data distribution, significance was assessed using the unpaired two-tailed *t*-test or one-way analysis of variance, as appropriate. Significant differences are indicated in the figures with * *p* < 0.05. All differences are considered statistically significant at *p* < 0.05, unless indicated otherwise. GraphPad Prism version 8.0 (GraphPad Software Co., Ltd, San Diego, CA, USA) was used for graphical illustrations and statistical analyses. The non-parametric factorial Kruskal–Wallis rank sum test was used to detect genera with significant abundance differences between groups, and the Wilcoxon rank sum test was then used to analyse the differences between the two groups. Finally, linear discriminant analysis was performed to achieve dimensionality reduction and assess the impact size of the significantly different genera.

## 5. Conclusions

We found that phorate can cause hyperglycaemia in mice. An in-depth analysis of the intestinal microbiota in mice revealed that phorate can affect the abundance of the intestinal microbiota and therefore alter the expression of drug-resistance genes. Moreover, changes in the abundance of the intestinal microbiota are closely related to the presence of antibiotic-resistant bacteria in the intestinal tract and the host’s metabolic health. Taken together, our results can guide pesticide safety evaluations in future studies.

## Data Availability

Not applicable.
